# Optimising antidepressant therapy in pregnancy: a perspective on therapeutic drug monitoring and model-informed precision dosing

**DOI:** 10.3389/fphar.2026.1817244

**Published:** 2026-04-30

**Authors:** Sílvia M. Illamola, Kathleen M. Job, Angela K. Birnbaum, Maged M. Costantine, Catherine M. Sherwin

**Affiliations:** 1 Department of Experimental and Clinical Pharmacology, College of Pharmacy, University of Minnesota, Minneapolis, MN, United States; 2 Division of Clinical Pharmacology, Department of Pediatrics, University of Utah School of Medicine, Salt Lake City, UT, United States; 3 Center for Clinical and Cognitive Neuropharmacology, College of Pharmacy, University of Minnesota, Minneapolis, MN, United States; 4 Department of Medical Laboratory Sciences, College of Pharmacy, University of Minnesota, Minneapolis, MN, United States; 5 Division of Maternal Fetal Medicine, Department of Obstetrics and Gynecology, The Ohio State University, Columbus, OH, United States; 6 Internal Medicine, UWA Medical School, The University of Western Australia, Perth, WA, Australia; 7 Differentia Biotech Ltd, South San Francisco, CA, United States; 8 Department of Pharmacology and Toxicology, Wright State University Boonshoft School of Medicine, Dayton, OH, United States

**Keywords:** antidepressants, clinical pharmacology, maternal foetal health, model-informed precision dosing, pharmacokinetics, pregnancy, SSRIs, therapeutic drug monitoring

## Abstract

Depression during pregnancy is common worldwide, with higher prevalences in low-income countries compared to high-income countries. Yet, current antidepressant dosing strategies in pregnant patients rarely account for the substantial pharmacokinetic shifts that occur. Altered hepatic metabolism and enhanced renal clearance can reduce plasma concentrations, potentially compromising efficacy. Therapeutic drug monitoring (TDM) offers one strategy: targeting plasma concentrations that correlate with adequate central nervous system (CNS) target engagement. Model-informed precision dosing (MIPD) takes this further, using population pharmacokinetic modelling to predict individualised doses. We examined prescribing patterns in an IRB-approved retrospective cohort and observed that dosing remained stable across trimesters despite reported pharmacokinetic alterations. This disconnect between emerging pharmacological knowledge and current practice deserves attention. Challenges remain such as assay availability is limited, there are fundamental cost considerations, and pregnancy-specific reference ranges need further development. Our position is that interdisciplinary collaboration should drive the growth of standardised protocols that integrate TDM and MIPD into prenatal care. Prospective trials will ultimately establish clinical benefit, but the mechanistic rationale already warrants systematic investigation.

## 1 Introduction

Major depressive disorder is common worldwide. The prevalence of depression among women of childbearing age in high-income countries ranges from 7% to 15% (Bennett et al., 2004; Molenaar et al., 2018). In contrast, the prevalence has been reported to be higher in low-income countries, with values from 22.7% to 47% (Hartley et al., 2011; Malqvist et al., 2016; Redinger et al., 2018; Rochat et al., 2011). The consequences of leaving it untreated extend beyond the mother: postpartum depression, impaired mother-infant bonding, preterm birth, and low birth weight (Dietz et al., 2007; Jarde et al., 2016; Previti et al., 2014). Antidepressants are commonly prescribed, but foetal exposure concerns create fundamental therapeutic uncertainty (Bulletins--Obstetrics, 2008; Rommel et al., 2022).

What does antidepressant efficacy actually require? From a neuropharmacological standpoint, sustained modulation of central serotonergic pathways requires adequate central nervous system (CNS) drug exposure. Positron emission tomography (PET) studies using [^11^C]DASB have shown that therapeutic doses of selective serotonin reuptake inhibitors (SSRIs) achieve approximately 80% striatal serotonin transporter (SERT) occupancy; the plasma concentration-occupancy relationship follows a hyperbolic curve (Hart et al., 2024; Meyer et al., 2004; Sorensen et al., 2022). Although reduced SERT occupancy plausibly increases relapse risk, the quantitative relationship between occupancy decline and clinical symptom recurrence during pregnancy remains insufficiently characterised. Additionally, pregnancy is associated with neuroendocrine and psychosocial factors that can modify antidepressant effectiveness in ways we do not fully understand. Therefore, SERT occupancy may serve as a mechanistic anchor for CNS target engagement, but not as a direct surrogate for clinical response.

Here is where pregnancy complicates things. The pharmacokinetic changes are profound and may compromise this therapeutic threshold, as seen with increased hepatic blood flow. Cytochrome P450 (CYP450) activity ramps up. Plasma volume expands. Renal clearance rises. Together, these shifts tend to reduce antidepressant concentrations (Koren and Pariente, 2018; Pariente et al., 2016; Tasnif et al., 2016). Sertraline, for instance, shows reduced dose-adjusted serum concentrations in late pregnancy; fluoxetine, citalopram, and venlafaxine follow similar patterns (Alenezi and Badhan, 2024; Freeman et al., 2008; Sit et al., 2008; Westin et al., 2017). The result can be subtherapeutic CNS exposure and increased relapse risk, or, with long-half-life agents like fluoxetine, the potential for supratherapeutic concentrations postpartum when clearance normalises if doses are not readjusted (Heikkinen et al., 2003). Elevated foetal exposure has been associated with neonatal adaptation syndrome (Grigoriadis et al., 2013).

There is another layer to consider: blood-brain barrier P-glycoprotein (P-gp). This transporter varies considerably among antidepressants. Venlafaxine, paroxetine, and tricyclics are P-gp substrates; fluoxetine and, to a lesser extent, citalopram penetrate the CNS more predictably (O'Brien et al., 2012; Uhr et al., 2007). For P-gp substrate antidepressants, pregnancy-induced changes in both systemic pharmacokinetics and transporter expression could compound CNS exposure alterations, which is precisely why clinical symptom monitoring should accompany TDM.

## 2 Illustrative prescribing patterns

**FIGURE 1:**
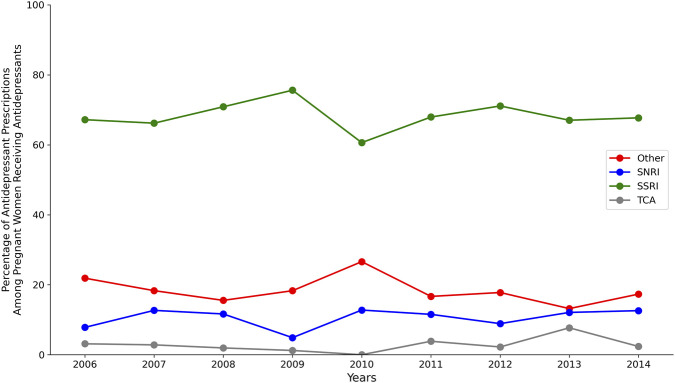
Trends in antidepressant prescribing among pregnant women receiving antidepressants (2006–2014). SSRIs (green) comprised 61%–76% of prescriptions, with a notable dip to 61% in 2010. SNRIs (blue) ranged from 5% to 13%. Other antidepressants (red) ranged from 13% to 27%, peaking in 2010. TCAs (grey) remained below 8%. Percentages calculated as class count divided by total prescriptions per year. See Methods for cohort details.

To contextualise current clinical practice, we examined prescribing patterns in an Institutional Review Board (IRB)-approved Institutional Review Boards of Intermountain Healthcare (IRB 1024250) and the University of Utah (IRB 00055665), de-identified retrospective cohort of 661 pregnant women aged 18–48 years who received antidepressants at Intermountain Healthcare and University of Utah facilities between 2006 and 2014. This descriptive analysis characterised aggregate prescribing trends rather than individual clinical outcomes; no TDM concentration data were available. Selective serotonin reuptake inhibitors (SSRIs) comprised 68% of prescriptions, with sertraline (28%), fluoxetine (16%), and citalopram (14%) being the most common among pregnant women prescribed an antidepressant. Serotonin-norepinephrine reuptake inhibitors (SNRIs) and bupropion each accounted for approximately 10% (Figure 1).

**FIGURE 2:**
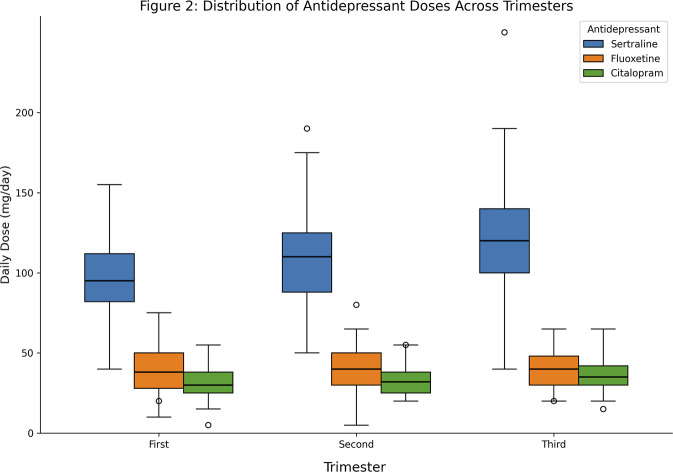
Distribution of daily doses across pregnancy trimesters for sertraline (blue), fluoxetine (orange), and citalopram (green). Box plots display median, interquartile range, whiskers (1.5× IQR), and outliers. No statistically significant differences were observed (Linear mixed effect model: p = 0.674 for mean daily dose; p = 0.549 for weight-adjusted dose). See Methods for cohort details. **Abbreviations:** SSRI, selective serotonin reuptake inhibitor; SNRI, serotonin-norepinephrine reuptake inhibitor; TCA, tricyclic antidepressant; IQR, interquartile range.

What stood out was the stability of antidepressant dosing across pregnancy. Mean daily antidepressant dose and dose per weight were compared across pregnancy trimesters and among different drugs using a linear mixed-effects model. The model included fixed effects for drug, pregnancy trimester, and their interaction, and a random effect for pregnancy nested within participants to account for within-pregnancy and within-participant correlation. The daily dose was log-transformed for analysis. An ANOVA test was used to assess the significance of the model’s fixed effects. Despite well-documented pharmacokinetic changes in the literature, no significant differences emerged in mean daily dose (p = 0.674) or weight-adjusted dose (p = 0.549) across trimesters. Weight-adjusted dosing was calculated using contemporaneous recorded weights within each trimester rather than the pre-pregnancy baseline weight. Looking at the three most commonly prescribed SSRIs, sertraline, fluoxetine, and citalopram, median doses stayed consistent from first through third trimester (Figure 2); though visual inspection suggested a modest upward trend for sertraline that did not reach statistical significance; only 20.4% of cases involved any dose adjustment. An alternative interpretation is that symptom control may have remained adequate in many patients despite pharmacokinetic changes, or that clinicians appropriately prioritised clinical stability over theoretical exposure targets in the absence of validated pregnancy-specific reference ranges.

This pattern of lack of dose adjustment in SSRI may point to a therapeutic gap, a disconnect between what pharmacology tells us and what happens in clinical practice. But we need to be careful about what these descriptive data can and cannot tell us. First, without clinical outcome measures, we have no way of knowing whether stable dosing led to a suboptimal therapeutic response. The retrospective design rules out causal inference. Second, the lack of specific diagnoses for which antidepressants may have been prescribed limits the ability to interpret stable doses as a potential therapeutic gap or as clinical evidence for disease-specific target attainment. These findings illustrate prescribing patterns; they do not demonstrate harm from current practice or prove benefit from doing things differently. To that end, we need prospective studies correlating concentrations with clinical outcomes.

## 3 Therapeutic drug monitoring: rationale and application

TDM is straightforward in concept: measure plasma concentrations and use them to guide dosing. The therapeutic reference ranges for sertraline (10–150 ng/mL), fluoxetine plus norfluoxetine (120–500 ng/mL), and citalopram (50–110 ng/mL) are mainly derived from nonpregnant populations and are extrapolated from SERT occupancy data (Hiemke et al., 2011; Hiemke et al., 2018; Johnson-Davis and Doyle, 2020; Uhr et al., 2007). TDM is not new for pregnancy-affected medications; lamotrigine TDM-guided adjustments are already standard practice to reduce seizure risk (Tran et al., 2002).

What might TDM offer during pregnancy? Several things, potentially. First, when symptoms recur, TDM helps distinguish pharmacokinetic underexposure from true pharmacodynamic non-response, a distinction that matters enormously for deciding between dose escalation and medication switching. Second, proactive adjustment becomes possible before clinical deterioration sets in. Third, TDM could identify supratherapeutic concentrations that may increase foetal exposure and the risk of neonatal adaptation syndrome (Grigoriadis et al., 2013). However, research on TDM during pregnancy is frequently limited by ethical and safety concerns regarding foetal exposure, leading to the underrepresentation of typical pregnancies in study populations. This limitation results in insufficient data to establish standardized TDM protocols for pregnant women (Johnson-Davis and Doyle, 2020). Additionally, physiological changes during pregnancy, such as increased renal clearance and altered drug metabolism, complicate the establishment of consistent TDM guidelines. Although some strategies for TDM during pregnancy have been suggested for antiepileptic drugs (Arfman et al., 2020), the variability in PK among pregnant women necessitates more extensive, more representative studies to develop effective TDM strategies (Moreira et al., 2023). Also, the limited number of large, randomized controlled trials means studies are often smaller and retrospective, limiting the ability to draw firm conclusions regarding the clinical utility of TDM use and improved outcomes. Therefore, the potential benefits of TDM still require validation through prospective studies.

### 3.1 Illustrative hypothetical case 1

Consider a composite scenario. A woman stable on sertraline 100 mg starts experiencing symptom recurrence in her second trimester. Without TDM, the assumption might be that the medication is no longer working. However, if TDM reveals subtherapeutic concentrations, this implicates pregnancy-related PK changes as the culprit rather than actual medication failure. That distinction guides a rational dose adjustment rather than an unnecessary switch to a different medication (Freeman et al., 2008; Paulzen et al., 2017; Stika et al., 2022).

### 3.2 Illustrative hypothetical case 2

The opposite scenario. A woman on fluoxetine 40 mg develops restlessness and insomnia in late pregnancy. If TDM shows supratherapeutic concentrations of fluoxetine and norfluoxetine, dose reduction makes sense, addressing her symptoms while potentially reducing foetal exposure. These composite cases illustrate how TDM might inform decision-making, though they obviously cannot establish generalizable efficacy (Heikkinen et al., 2003; Sit et al., 2008).

## 4 Model-informed precision dosing

MIPD takes TDM a step further. It integrates population pharmacokinetic models with patient-specific factors, such as gestational age, weight, and CYP genotype, to predict optimal dosing. The approach uses Bayesian estimation, combining prior information about pregnancy pharmacokinetics with individual characteristics and measured concentrations to generate personalised recommendations (Faelens et al., 2022; Hughes et al., 2021). Published population models already characterise how pregnancy changes antidepressant disposition, and these could inform starting doses and help predict when adjustments will be needed (George et al., 2020; Westin et al., 2017; Yue et al., 2023).

MIPD becomes particularly valuable when TDM access is limited, which describes most clinical settings. Population models can guide empirical adjustments based on gestational age and patient characteristics even without measured concentrations. Web-based platforms with user-friendly interfaces are emerging, allowing clinicians without pharmacometrics expertise to access dose-optimisation tools (Moreira et al., 2023; Preijers et al., 2024). However, model validation in diverse obstetric populations remains limited, and we still need clinical outcome studies.

## 5 Implementation challenges and future directions

Barriers to the routine implementation of TDM and MIPD are real. On the practical side: there is a need for clinician education, limited assay availability, monitoring costs, specialised infrastructure requirements, and inconsistent reimbursement. On the evidence side, pregnancy-specific therapeutic ranges remain poorly defined, and randomised trials comparing TDM-guided care with standard care do not yet exist. The relationship between maternal concentrations, placental transfer, and foetal exposure adds another layer of complexity. TDM and MIPD have the potential to improve dose individualisation in response to dynamic physiological changes, minimising risks to both mother and child. However, actualising this potential requires overcoming significant logistical and clinical hurdles. Therefore, we advocate for robust research initiatives and comprehensive policy development to support the standardisation of TDM and MIPD practices.

One pragmatic approach is to prioritise TDM for high-risk patients, those with treatment-resistant depression, significant pharmacogenetic variability, complex polypharmacy, or symptom recurrence during pregnancy. Symptom-triggered TDM offers an intermediate strategy: obtain concentrations when symptoms worsen despite stable dosing. As for broader adoption, emerging technologies may help. Point-of-care testing, dried blood spot sampling, and telemedicine integration are methodologies that continue to evolve and could make TDM more accessible.

What research do we need? Prospective studies correlating concentrations with clinical outcomes. Pregnancy-specific reference ranges. Comparative effectiveness trials. Pharmacogenomic integration deserves attention, too; combining TDM with genotype-guided dosing could prove valuable (Almurjan et al., 2021; Colombo et al., 2021). And if TDM is going to matter clinically, we will need to address disparities in access across geographic and socioeconomic contexts where maternal depression is especially prevalent. Socioeconomic factors, geographic location, differences in healthcare systems, and the high cost of TDM and MIPD may limit the overall implementation into the clinical practice.

## 6 Author perspective

Our perspective is straightforward: current antidepressant management during pregnancy likely fails to account for pharmacokinetic variability. The prescribing patterns we observed tell a story of stable dosing despite pharmacokinetic changes that are far from stable. Does this disconnect between pharmacological knowledge and clinical practice cause harm? We cannot conclude solely from descriptive data. But the mechanistic rationale for individualised dosing is difficult to ignore pregnancy-induced pharmacokinetic changes are substantial, the concentration-occupancy relationship is well characterised, and we already accept TDM for lamotrigine in this population.

What would help? An interdisciplinary collaboration among clinical pharmacologists, psychiatrists, obstetricians, and laboratory specialists to develop standardised TDM protocols would be beneficial. Professional societies should provide consensus recommendations on indications, timing, and therapeutic targets. For clinicians seeing patients now, we suggest screening for symptom recurrence each trimester, considering symptom-triggered TDM when the response is inadequate, and maintaining plasma concentrations at the patient’s known pre-pregnancy therapeutic level if such data are available.

Bridging this therapeutic gap could improve maternal mental health while optimising foetal safety, or at least that is the hypothesis worth testing. TDM and MIPD are tools; their clinical value in pregnancy needs systematic validation. The neuropharmacological rationale holds up, and the implementation pathway is reasonably straightforward. What is missing is rigorous research demonstrating that these approaches translate into meaningful clinical benefits.

## Data Availability

Summary statistics are available upon reasonable request from the corresponding author, subject to institutional approvals. Requests to access these datasets should be directed to Catherine M. Sherwin, PhD, MD. Email: Catherine.sherwin85@gmail.com.
